# Drug-Induced Pemphigus Foliaceus Potentially Triggered by Piperacillin-Tazobactam, Linezolid, and Additional Factors: A Report of a Rare Case

**DOI:** 10.7759/cureus.85144

**Published:** 2025-05-31

**Authors:** Aishwarya Holi, Satya Rijal, Sumugdha Rayamajhi, Nikhil Regmi

**Affiliations:** 1 Internal Medicine, Michigan State University College of Human Medicine, East Lansing, USA

**Keywords:** bullous skin lesions, desmoglein, drug-induced pemphigus, pemphigus foliaceous, pemphigus triggers

## Abstract

Drug-induced pemphigus (DIP) is a rare autoimmune blistering disorder most commonly associated with thiol-containing drugs such as penicillamine, captopril, and bucillamine. However, additional medications are increasingly recognized in the nonthiol nonphenol group. While some cases require treatment with corticosteroids or immunosuppressants, in certain instances, simply discontinuing the offending drug is sufficient. We report a unique case of drug-induced pemphigus foliaceus (DIPF) potentially triggered by piperacillin-tazobactam and/or linezolid in a 69-year-old female patient on the 10th day of hospitalization while receiving treatment for necrotizing fasciitis and *Streptococcus agalactiae* bacteremia. To the best of our knowledge, this is the first reported case of DIP caused by piperacillin-tazobactam and/or linezolid. However, other factors may have predisposed them. The diagnosis of DIPF was confirmed through skin biopsy, direct immunofluorescence (DIF), and enzyme-linked immunosorbent assay (ELISA). Early recognition and prompt discontinuation of the suspected drugs likely prevented disease progression, emphasizing the importance of vigilance when managing patients on polypharmacy. While the exact mechanism of DIP remains unclear, potential pathways include antigen modification, immune dysregulation, or oxidative stress. This case highlights the need for heightened awareness of atypical triggers, comprehensive diagnostics, and timely intervention to optimize patient outcomes.

## Introduction

Pemphigus foliaceus (PF) belongs to a group of bullous autoimmune skin diseases caused by autoantibodies against adhesion molecules, leading to fragile blisters and erosions. Drug-induced pemphigus (DIP) is rare, accounting for 10% of cases. PF is the most common presentation of DIP, occurring in up to 70% of thiol-induced cases, while a nonthiol group of drugs usually tends to manifest as pemphigus vulgaris (PV) [1]. The etiology behind the development of DIP is not well understood but is related to the breakage of central and peripheral immune tolerance. This immune imbalance may be due to predisposing genetic and external factors. Human leukocyte antigen-DR beta 1 (HLA-DRB1) polymorphisms are a known significant risk factor for PV. In addition, single-nucleotide polymorphisms (SNPs) are used as a marker for predicting treatment response and disease prognosis [2].

Genetic and external factors influence the development of pemphigus. However, low concordance rates in monozygotic twins and the rarity of case reports of familial cases of pemphigus highlight that genetics alone is insufficient for disease onset. Environmental factors further influence outcomes, as studies show many first-degree relatives of pemphigus patients have autoantibodies without disease due to protective genetics or environmental avoidance [3].

## Case presentation

A 69-year-old woman with uncontrolled type 2 diabetes mellitus was admitted for necrotizing fasciitis of the right lower extremity, requiring a below-knee amputation on day 1. She also had *Streptococcus agalactiae* bacteremia and was initially treated with intravenous (IV) cefepime, vancomycin, and clindamycin, later narrowed to IV cefazolin for a two-week course. On day 6, she was transferred to inpatient rehabilitation, and IV cefazolin was switched to oral linezolid.

**Figure 1:**
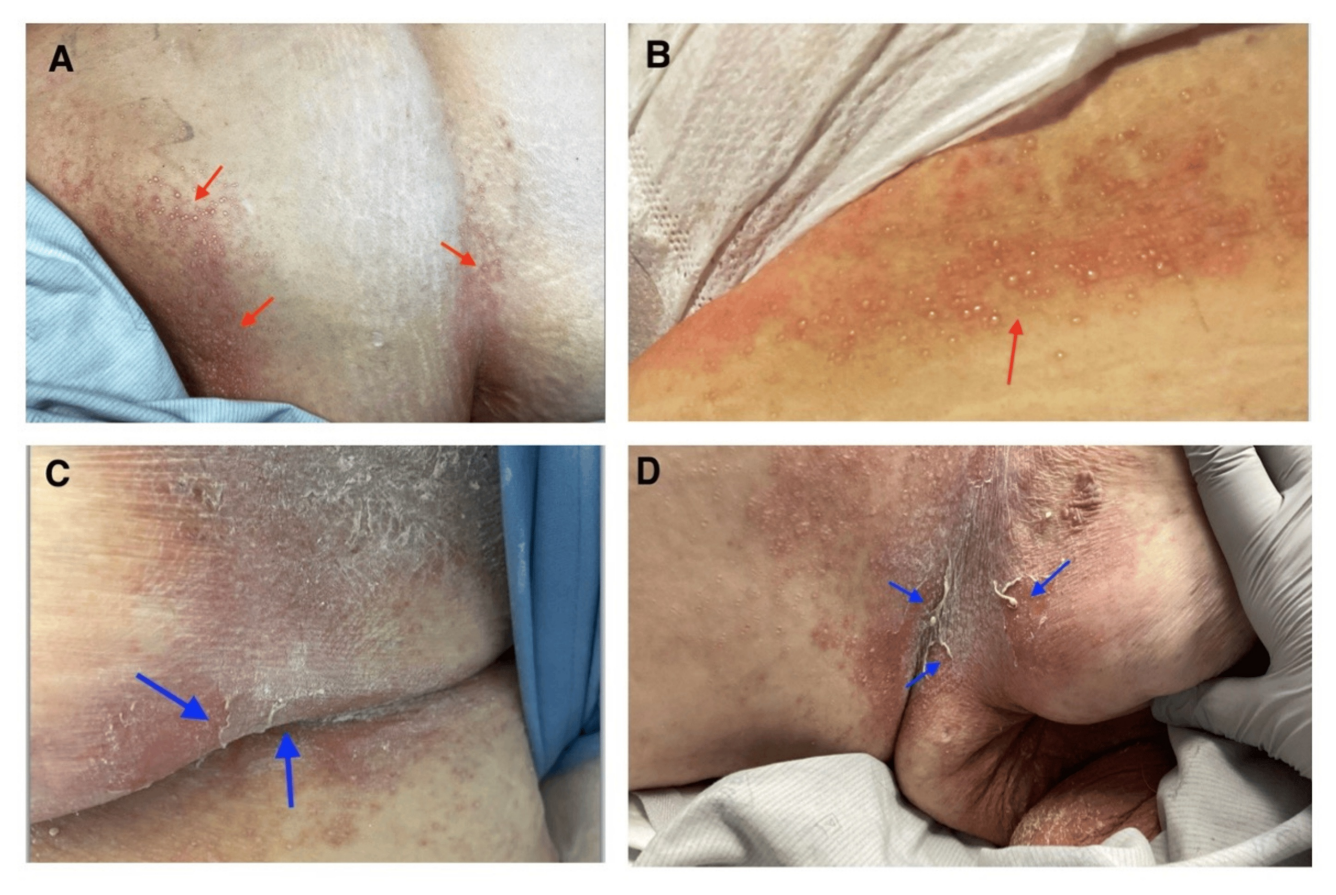
Clinical images of pemphigus foliaceus (A, B) Multiple small vesiculobullous lesions on the right breast, right inframammary fold, and trunk (red arrows). (C, D) "Puff pastry-like" lesions on the left breast and left inframammary fold following rupture of vesiculobullous lesions (blue arrows).

On day 7, worsening stump pain, leukocytosis, and serosanguineous discharge from the stump led to the addition of IV piperacillin-tazobactam. By day 10, she developed painful, non-pruritic skin lesions with burning and tingling, presenting as flaccid vesicles and bullous erosions that ruptured into erythematous, scaly plaques on the trunk, back, and inframammary folds, sparing mucosal surfaces (Figures 1A-1D).

Her past medical history included osteoarthritis, type 2 diabetes mellitus, and hypertension. Her home medications consisted of acetaminophen 500 mg every eight hours as needed, lisinopril 20 mg daily, metformin 1000 mg daily, and insulin glargine 15 units nightly. She had no personal or family history of rheumatological disease and reported no known drug or environmental allergies.

The laboratory findings on day 7 revealed elevated white blood cell (WBC), absolute neutrophil count (ANC), C-reactive protein (CRP), and erythrocyte sedimentation rate (ESR) of 21.4 (×10³/µL), 15.60 (×10³/µL), 16 (mg/dL), and 52 (mm/h), respectively, compared to day 1 (Table 1).

**Table 1:** The relevant laboratory findings on day 1 and day 7 ANC: absolute neutrophil count; CRP: C-reactive protein; ESR: erythrocyte sedimentation rate; HSV: herpes simplex virus; VZV: varicella-zoster virus; PCR: polymerase chain reaction; IgG: immunoglobulin G

Test	Day 1	Day 7	Reference range	Other results
WBC	14.3	21.4	4.0-12.0 (×10³/µL)	-
Hemoglobin	9.6	9.8	12.0-15.0 (g/dL)	-
Neutrophils	91.5	82.7	49.0-81.0 (%)	-
ANC	13.10	15.60	1.96-9.72 (×10³/µL)	-
CRP	14.7	16	0.0-1.0 (mg/dL)	-
ESR	30	52	0-20 (mm/h)	-
HSV I, II, VZV (PCR)	-	-	Not detected	Not detected
Desmoglein 1 IgG	-	-	<20 (RU/mL)	43.2 (positive)
Desmoglein 3 IgG	-	-	<20 (RU/mL)	11.1 (negative)

**Figure 2:**
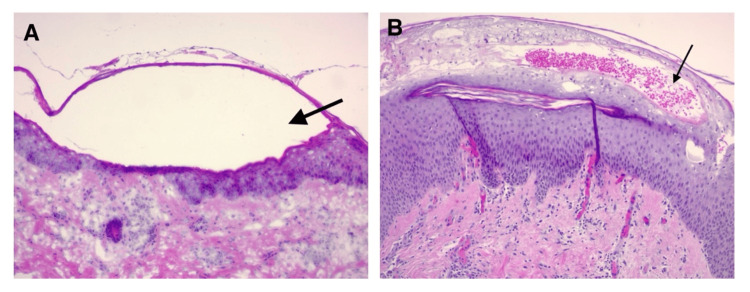
A skin biopsy with H&E staining (A) Subcorneal blister with clear fluid and superficial mononuclear cell infiltrate and papillary dermal edema. (B) Hyperplastic epidermis with intraepidermal and subcorneal blister and occasional neutrophils, eosinophils, and scattered melanophages. Black arrows indicate sub-corneal blisters. H&E: hematoxylin and eosin

A skin biopsy from the abdominal lesions with hematoxylin and eosin (H&E) staining showed a slightly hyperplastic epidermis with clear fluid-filled blisters in the stratum corneum, superficial mononuclear cell infiltrate, and papillary dermal edema (Figures 2A-2B). DIF testing showed strong positivity for IgG (++++/++++) and C3 (++++/++++) in the rim of the blister and intercellular bridge of the adjacent keratinocytes. The enzyme-linked immunosorbent assay (ELISA) test showed a weakly positive result for desmoglein 1 (Dsg1) IgG antibody at 43.2 RU/mL (normal: <20 RU/mL) and a negative result for desmoglein 3 (Dsg3) IgG antibody at 11.1 RU/mL (normal: <20 RU/mL). Polymerase chain reaction (PCR) testing was negative for herpes simplex virus (HSV) I, II, and varicella zoster virus (VZV) (Table 1).

Suspicion of drug-induced etiology arose three to four days after the onset of skin lesions. Piperacillin-tazobactam was stopped first, followed by linezolid two days later when no improvement was seen. By this time, the patient had completed a two-week course for *S. agalactiae *bacteremia. Due to concerns about a right stump infection, IV aztreonam was started for gram-negative coverage per infectious disease recommendations and continued for three days, completing a seven-day course.

Throughout the course, the patient received local skin care with emollients, and lesions were left open to the air to promote healing. Notably, no systemic or topical steroids or immunosuppressive agents were administered. Skin lesions began improving by day 6 after discontinuing antibiotics, with complete resolution within three months.

## Discussion

The clinical features of DIP resemble those of idiopathic pemphigus, with PF being the most common subtype. PF is characterized by multiple fragile, superficial blisters that rupture easily, resulting in erythematous, scaly, crusted plaques and erosions. The most common areas affected are the scalp, face, and upper trunk, typically following a seborrheic distribution [1]. The skin lesions in our patient differ from usual cases as they were located on the trunk, back, and inframammary folds.

Unlike PV, PF does not involve oral mucosa and is limited to cutaneous manifestations, usually presenting as puff pastry-like scale formation. The average incubation period for DIP typically ranges from a few weeks to two months. However, there have been documented cases with incubation periods extending up to six years (with anticonvulsants) to much shorter latency periods with repeated exposure [1,2].

IgG autoantibodies against Dsg 3 and Dsg 1 are hallmarks of mucocutaneous PV, while autoantibodies against Dsg 1 are the hallmarks of PF [4]. In this case, despite the weakly positive Dsg1 antibody, the clinical presentation and skin biopsy findings strongly support PF as the diagnosis.

The histopathology in drug-induced pemphigus foliaceus (DIPF) is characterized by superficial intradermal cleavage with acantholysis beneath the stratum corneum or granular layer. Additional findings may include a mixed inflammatory infiltrate in the dermis predominantly composed of eosinophils and eosinophilic spongiosis. Direct immunofluorescence (DIF) often shows intercellular deposition of IgG in the epidermis, with or without C3, present in up to 90% of DIP cases. Serological studies like indirect immunofluorescence (IIF) and ELISA aid diagnosis, with ELISA being highly sensitive and specific, useful when IIF is negative [5].

The potential differential diagnoses for generalized vesiculobullous diseases are Stevens-Johnson syndrome, staphylococcal scalded skin syndrome, toxic epidermal necrolysis, PV, and paraneoplastic pemphigus. However, these conditions usually involve mucosal surfaces and present with acute illness, which our patient did not exhibit. Furthermore, the ELISA test was positive only for the Dsg1 antibody, indicating a diagnosis of PF rather than PV (Table 1).

Bullous impetigo is a potential differential diagnosis due to overlapping clinical and histological features with PF. However, it is caused by *Staphylococcus aureus* or group A *Streptococcus*, while our patient had *S. agalactiae* (group B). The lesions appeared 3-4 days after starting piperacillin-tazobactam and linezolid, an atypical timeline, and took three months to resolve, unlike the rapid resolution seen in bullous impetigo. Skin biopsy showed no gram-positive organisms, and DIF was consistent with PF, making bullous impetigo unlikely.

Other possible differential diagnoses include IgA dermatosis, disseminated herpes simplex infection, herpes zoster, and allergic contact dermatitis. The absence of IgA deposition along the dermal-epidermal junction on DIF testing, along with negative serologies for HSV I/II and VZV (Table 1), excluded IgA dermatosis, disseminated herpes simplex infection, and herpes zoster. Allergic contact dermatitis typically appears in localized areas that have been exposed to allergens; however, diffuse dermatitis can occur if the allergen affects larger body regions, such as with textile dyes. In our case, the results from skin biopsy (Figures 2A-2B), DIF, and ELISA tests supported a diagnosis of PF over allergic contact dermatitis (Table 1).

Table 2 outlines factors identified as potential triggers for pemphigus across different categories.

**Table 2:** Known triggers of pemphigus Credit: Adapted and modified from reference [6], under the terms of the Creative Commons Attribution 4.0 International License (CC BY 4.0).

Category	Triggers
Drugs	Angiotensin-converting enzyme (ACE) inhibitors: cilazapril, fosinopril, lisinopril, captopril, enalapril, benazepril, quinapril, ramipril
	Non-steroidal anti-inflammatory drugs (NSAIDs): acetylsalicylic acid, metamizole
	Angiotensin receptor blockers: losartan, irbesartan
	Calcium channel blockers: nifedipine
	Biologic drugs: secukinumab, tocilizumab
	Other drugs: imiquimod, carbamazepine, glibenclamide, ceftazidime, hydrochlorothiazide, hydroxychloroquine, ingenol mebutate, rifampin, levodopa, heroin, penicillamine, gold sodium thiomalate, penicillin, bucillamine, oral contraception, 5-aminolaevulinic acid-based photodynamic therapy
Vaccines	Influenza, hepatitis B, rabies, tetanus, COVID-19 vaccines (Comirnaty, Vaxzevria, Spikevax, ChAdOx1 nCoV-19, Sinopharm COVID-19)
Infections	Viruses: human herpesvirus (HHV, HHV8), herpes simplex virus, cytomegalovirus, Epstein-Barr virus, hepatitis B virus, hepatitis C virus, human immunodeficiency virus (HIV), rotavirus
	Bacteria: *Helicobacter pylori *
Nutrition	Thiols: garlic, leek, chives, onion, shallot
	Phenols: mango, cashew nuts, black peppers, red chilies
	Tannins: mango, cassava, yucca, guarana, betel nut, raspberry, cranberry, blackberry, avocado, peach, ginger, tea, ginseng, red wine
	Isothiocyanates: mustard oil
	Phycocyanin: walnut proteins
Other factors	Pregnancy, radiation, emotional stress, pesticides (organophosphates, organochlorines), trauma (surgery, accidental traumas, electrical injury, bee sting), chemicals (photographic developing, dry cleaning, industrial solvents, phenol)

Drugs involved in DIP can be classified into three categories: i) thiol or sulfhydryl (-SH) group; ii) phenol group; and iii) nonthiol nonphenol group. Thiol or sulfhydryl (-SH) groups induce pemphigus by disrupting keratinocyte adhesion, forming thiol-cysteine bonds, and generating neoantigens (e.g., captopril and penicillamine) [7]. Phenol groups trigger pemphigus by releasing cytokines such as tumor necrosis factor-alpha (TNF-α) and interleukin-1 alpha (IL-1α) and activating the complement system and proteases, causing acantholysis (e.g., aspirin, rifampin) [8]. Nonthiol nonphenol drugs induce pemphigus through their active amide groups, though the underlying process remains unknown (e.g., pyrazolone derivatives, and enalapril) [9].

The mechanisms by which piperacillin-tazobactam and linezolid could have induced pemphigus in our patient are unclear, as their classification into thiol, phenol, or nonthiol nonphenol groups is uncertain. However, the development of vesiculobullous lesions shortly after exposure, followed by spontaneous resolution upon discontinuation, suggests that one or both drugs may have contributed, though the exact culprit remains undetermined.

A review of the patient's medication list revealed chronic lisinopril use for hypertension, a drug known to induce pemphigus (Table 2). Nevertheless, as the skin lesions resolved after discontinuing piperacillin-tazobactam and linezolid while lisinopril was continued, it is unlikely that lisinopril was the primary trigger.

As shown in Table 2, our patient had three additional potential triggers of pemphigus: infection, surgery (right below-knee amputation), and emotional stress from life-altering surgery, suggesting the existence of a complex interplay of factors that may have predisposed the patient to the development of the disease. While no cases have been linked specifically to piperacillin-tazobactam or linezolid, penicillin-induced pemphigus is well-documented. Given piperacillin’s beta-lactam structure, cross-reactivity or another unknown mechanism cannot be ruled out. Although definitive evidence is lacking, the temporal correlation raises the possibility of a drug-induced mechanism, potentially compounded by infection, surgery, and emotional stress.

## Conclusions

DIP presents a significant diagnostic challenge because there are no distinct clinical, histological, or immunological features that differentiate it from idiopathic pemphigus. Therefore, it is crucial to consider DIP as a potential diagnosis in all new cases of pemphigus. This case highlights the importance of recognizing DIPF, especially in patients who are on multiple medications. Taking a detailed drug history is essential for identifying potential triggers. It is important for clinicians to be aware that medications such as piperacillin-tazobactam and linezolid may cause PF. The resolution of lesions after discontinuing the drug, without the need for immunosuppressive therapy, suggests a favorable prognosis. Further research is needed to explore the underlying mechanisms and to confirm these associations.
